# Amplified genome editing by *in vivo* editor production

**DOI:** 10.64898/2026.01.13.699115

**Published:** 2026-01-13

**Authors:** Wayne Ngo, Daniel Rosas-Rivera, Kevin M. Wasko, Longhui Qiu, Min Hyung Kang, Shaan Gogna, Jingkun Zeng, Mason T. Hooks, Jamie L.Y. Wu, Zhongmei Li, Jennifer A. Doudna

**Affiliations:** 1 Innovative Genomics Institute; University of California, Berkeley; Berkeley CA, USA.; 2 Gladstone Institute of Data Science and Biotechnology; San Francisco, CA, USA.; 3 California Institute for Quantitative Biosciences, University of California, Berkeley; Berkeley, CA, USA.; 4 Department of Molecular and Cell Biology, University of California, Berkeley; Berkeley, CA, USA.; 5 Department of Medicine, University of California, San Francisco; San Francisco, CA, USA.; 6 Gladstone-UCSF Institute of Genomic Immunology; San Francisco, CA, USA.; 7 Howard Hughes Medical Institute, University of California, Berkeley; Berkeley CA, USA.; 8 Molecular Biophysics and Integrated Bioimaging Division, Lawrence Berkeley National Laboratory; Berkeley, CA, USA.; 9 Department of Chemistry, University of California, Berkeley; Berkeley, CA, USA.; 10 Li Ka Shing Center for Genomic Engineering, University of California, Berkeley, Berkeley, CA, USA.

## Abstract

Genome editing enzymes have vast therapeutic potential. However, achieving sufficient delivery *in vivo* remains a major challenge, because editing machinery is confined to the subset of transfectable cells in a tissue. Here, we tested the possibility that genome editing could be amplified *in vivo* by programming transfected cells to produce and transfer editing enzymes in lipid vesicles to neighboring cells. Our data show that this NANoparticle-Induced Transfer of Enzyme (NANITE) strategy tripled editing efficiency in cultured cells relative to non-spreading controls. Furthermore, a single intravenous injection of the NANITE plasmid into mice induced ~3-fold higher levels of liver editing at the *Ttr* locus relative to non-spreading controls, with corresponding reductions in serum transthyretin levels. Amplifying therapeutic enzymes *in situ* offers a nonviral and non-infectious strategy to overcome low delivery efficiencies and reduce effective dose requirements.

Genome editing enzymes promise to revolutionize medicine by overriding or correcting the underlying genetic basis of disease. Successful treatment requires editing a sufficient fraction of target cells to exceed disease-specific therapeutic thresholds. For example, approximately 20% hematopoietic stem cells need to be corrected to ameliorate sickle cell disease^1^, while >15% dystrophin restoration is necessary to protect muscle function in Duchenne muscular dystrophy^2^. However, once delivered to cells, editing machinery is confined to the cells it initially enters. Because editing machinery cannot spread between cells, achieving these thresholds depends entirely on delivering enzymes to a sufficient fraction of cells. We wondered whether it is possible to engineer editing enzymes to transfer between cells following transfection, thereby amplifying therapeutic effects beyond initially transfected populations.

Cell-to-cell transfer of proteins and RNA has been demonstrated through natural mechanisms including exosomes^3^ and tunneling nanotubes^4^. Engineered systems have achieved transfer of proteins and RNA *in vitro*^5,6^ and *in vivo*^7–10^ using vesicles. However, no approach has yet enabled *in vivo* transfer of protein-RNA complexes such as CRISPR-Cas9 ribonucleoproteins (RNPs) from one cell to another. We hypothesized that equipping cells with components to package and transfer editing enzymes to neighboring cells could amplify genome editing well beyond the originally transfected population. We previously developed enveloped delivery vehicles (EDVs), virally-derived lipid vesicles that package *Streptococcus pyogenes* Cas9 (SpCas9) RNPs^11–13^. Following transfection, producer cells package and secrete SpCas9 RNPs in lipid vesicles displaying fusogens and targeting moieties on their surface. Harvesting, purifying and administering the EDVs to recipient cells *in vitro* and *in vivo* allows genome editing^11–13^. We wondered if enabling cells to transiently produce EDVs *in situ* could amplify editing *in vivo*.

Here, we amplified genome editing *in vivo* by locally producing EDVs to make and transfer SpCas9 RNPs following initial nucleic acid delivery (Fig. 1a). This NANoparticle Induced Transfer of Enzymes (NANITE) technology allows transfer of packaged editing enzymes between cells via non-replicative vesicles produced by initially transfected cells. Transfecting cells with a plasmid encoding the NANITE components amplified genome editing efficiencies by ~3-fold both *in vitro* and *in vivo* relative to non-spreading controls. NANITE thus provides a strategy to amplify therapeutic genome editing after delivery, lowering the delivery threshold required for therapeutic genome editing.

## Results

### Encoding NANITE components in a single plasmid

**Figure 1.**
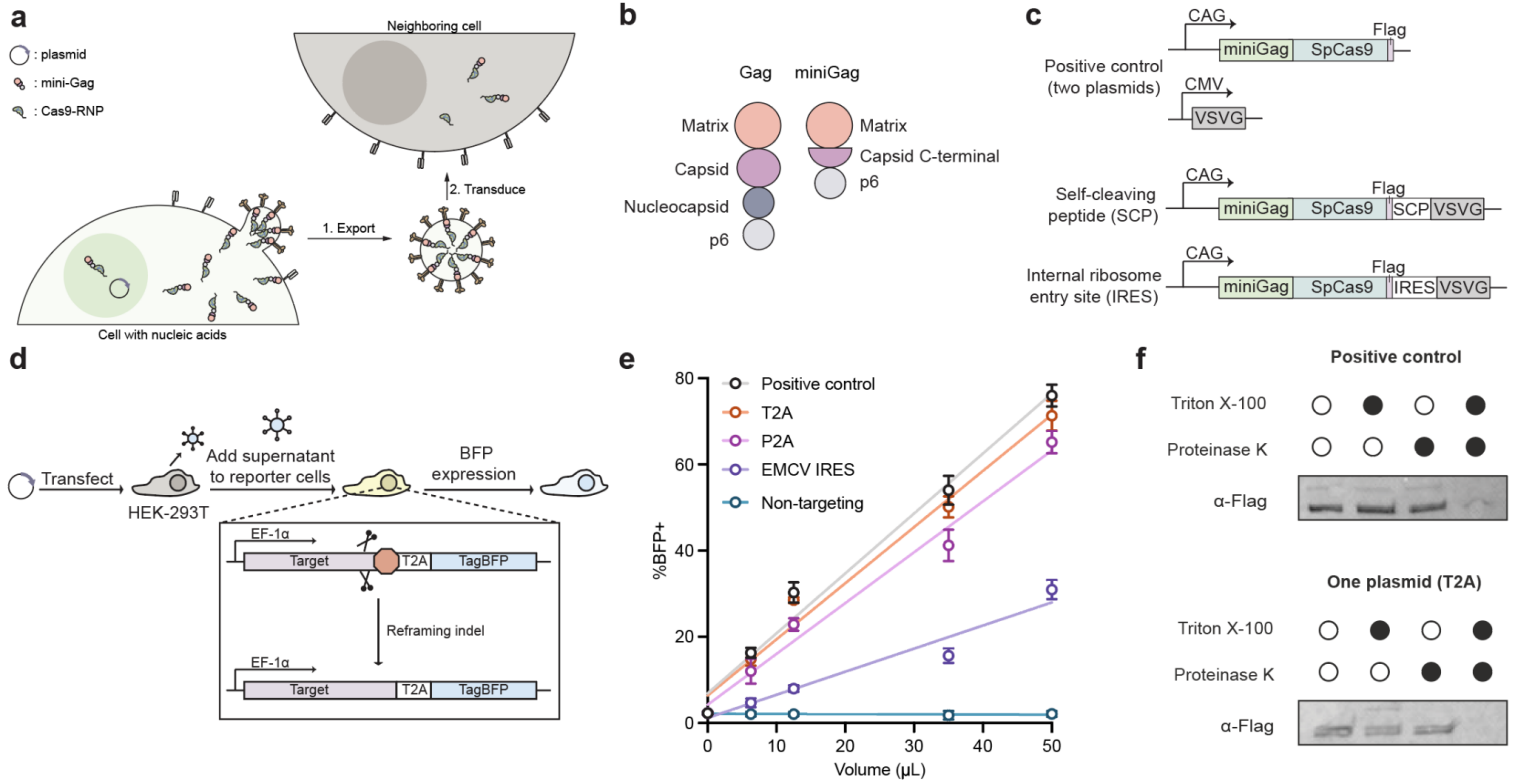
Genome editing vesicles can be produced from a single plasmid. **(a)** Schematic of the NANITE system, in which cells that receive nucleic acid cargo become factories that transiently produce and secrete Cas9 ribonucleoproteins in vesicles. Locally produced vesicles subsequently transduce neighboring cells to spread genome editing. **(b)** Schematic of the miniGag vesicle structural protein compared to HIV-1 Gag structural protein. **(c)** Design of NANITE. The positive control consists of the current two-plasmid system to produce miniEDVs, in which one plasmid encodes the cargo and internal structural components of the vesicle and a second plasmid encodes the surface protein necessary for cell transduction. In NANITE, the surface protein is encoded on the first plasmid, separated by either a self-cleaving peptide or an internal ribosome entry site. **(d)** Experimental scheme for testing NANITE constructs compared to the positive control. HEK-293T producer cells were transfected with plasmids. Supernatant containing EDVs was harvested 48 hours post-transfection and incubated with reporter cells that express blue fluorescent protein (BFP) upon genome editing. Schematics (a–d) are not to scale. **(e)** Fraction of reporter cells expressing BFP after incubation with supernatants, quantified by flow cytometry. Separating the surface protein from internal components using a T2A sequence resulted in EDVs with comparable activity to the positive control. Data are presented as mean ± SD of three replicates. **(f)** Protease protection assay of vesicles produced by either the positive control or T2A system. A FLAG tag is present on the C-terminus of Cas9, allowing internal components of the vesicle to be detected by anti-FLAG antibodies. Cas9 is enclosed within vesicles in both conditions, as both Triton X-100 and proteinase K are required for degradation. The assay was repeated twice with similar results.

We previously showed that fusing SpCas9 RNPs to truncated lentiviral Gag structural proteins (“miniGag”, Fig. 1b) allows cells to secrete EDVs that encapsulate editing enzymes^13^. These particles are non-replicative because they do not package nucleic acids encoding their components^13^. Although EDVs and virus-like particles (VLPs) have been generated *in vitro* using producer cells, we wanted to explore whether transient *in vivo* production could amplify genome editing effects. To explore this possibility, we first addressed the complexity of production which required co-delivery of multiple plasmids at fixed ratios that are difficult to control *in vivo*^11–15^. We designed single plasmids to express all the necessary components to assemble and secrete SpCas9 RNP-containing EDVs (Fig. 1c). Single-guide RNAs (sgRNAs) were encoded on the same plasmid and expressed from a U6 promoter. To test for EDV production, plasmids were transfected into HEK-293T cells, and the supernatant was harvested and incubated with reporter cells that express BFP upon genome editing (Fig. 1d and Supplementary Fig. 1a-c). All single plasmid constructs produced genome editing-competent EDVs as detected by flow cytometry (Fig. 1e, flow gating shown in Supplementary Fig. 1d, e). The T2A design performed nearly identically to the multi-plasmid positive control, while the P2A and encephalomyocarditis virus internal ribosomal entry site (IRES) designs showed reduced activity. Western blot analysis of the cell lysates confirmed complete T2A-catalyzed post-translational cleavage of the fusogen VSVG, with miniGag-Cas9 and VSVG detected at expected sizes (~210 kDa and 55 kDa) (Supplementary Fig. 2). A protease protection assay showed that SpCas9 RNPs were packaged inside vesicles, because internal components were protected from proteinase K degradation unless Triton X-100 disrupted the membrane (Fig. 1f). These results demonstrate that in contrast to prior EDV expression formats requiring three to six plasmids^11–13^, a single plasmid can encode fully functional genome editing EDVs. This simplifies EDV production both *in vitro* and *in vivo*. We used the T2A design for subsequent experiments, which we named “NANoparticle Induced Transfer of Enzymes (NANITE)”.

### NANITE amplifies editing in situ

**Figure 2.**
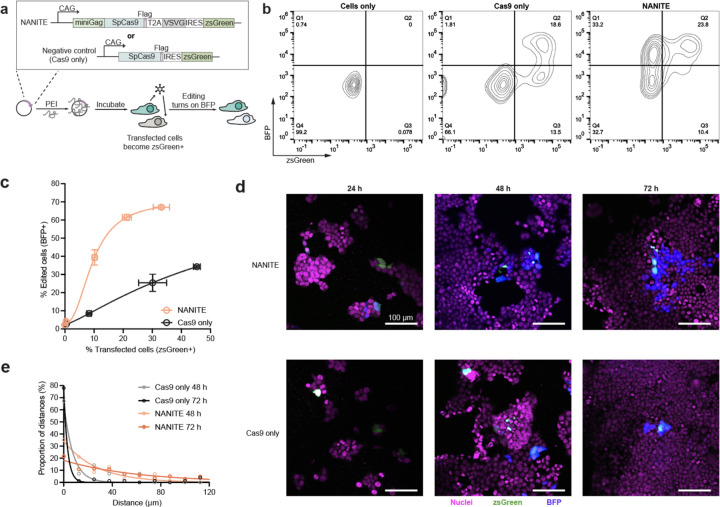
NANITE spreads genome editing *in situ*. **(a)** Experimental scheme for testing NANITE spread. Plasmids were complexed with polyethylenimine and incubated with reporter HEK-293T cells. Transfected cells express zsGreen. Genome editing restores BFP expression. Schematic is not to scale. **(b)** Representative flow cytometry plots showing zsGreen and BFP expression. Transfection with NANITE resulted in a population of cells that were edited (BFP^⁺^) but not transfected (zsGreen^−^). **(c)** Fraction of edited cells relative to transfected cells following transfection with NANITE or Cas9. More edited cells than transfected cells were observed in the NANITE condition. Data are presented as mean ± SD of three replicates. **(d)** Representative confocal microscopy images of cells transfected with NANITE or Cas9 plasmids. NANITE-transfected cells edit neighboring cells. Scale bar as indicated. Two biologicals were performed. **(e)** Distribution of distances between edited (BFP^⁺^) and nearest transfected (zsGreen^⁺^) cells, fitted to an exponential decay. Six fields of view from two biological replicates were analyzed. NANITE increases the distance between edited cells and their nearest transfected cell, indicating spread of genome editing.

We next explored whether *in situ* production of EDVs would allow editing enzymes to spread from transfected to untransfected cells. We added an IRES-zsGreen cassette to the NANITE plasmid for labeling transfected cells and designed a Cas9 RNP-only control (Fig. 2a). Plasmids were complexed with polyethylenimine and transfected into BFP reporter cells ^16,17^. Excess plasmids were removed after 24 hours, and transfected (zsGreen+) and edited (BFP+) cells were quantified by flow cytometry the following day (Fig. 2b, gating in Supplementary Fig. 1d). Transfecting cells with Cas9 alone yielded 32% transfected (zsGreen+) and 19% edited (BFP+) cells. All edited cells were transfected, but only a subfraction of the transfected cells were edited due to either insufficient Cas9 production or indels that did not restore BFP expression (Supplementary Fig. 1a). In contrast, transfecting cells with NANITE produced 34% transfected and 57% edited cells. Notably, 33% of cells were edited without transfection (BFP+, zsGreen−) (Fig. 2b). Across transfection efficiencies, only ~87% of cells transfected with Cas9 plasmids became edited, while NANITE-edited cells averaged 293% of the transfected population with a maximum of 386% (Fig. 2c). These results represent a departure from prior work, where the effects of editing enzymes and nucleic acid cargos are typically confined to transfected cells. NANITE enables their spread to untransfected neighbors.

To visualize the extent of editing over time, we imaged cells after low-dose transfection to observe discrete spreading events (Fig. 2d). Transfected zsGreen+ cells appeared within 24 hours in both Cas9 and NANITE conditions. BFP+ edited cells emerged after 48 hours. We observed clusters of edited cells surrounding cells transfected with NANITE. To quantify the distribution of spread, we measured distances from each edited cell to the nearest transfected cell center-to-center using Cellprofiler^18^. These distances were binned into histograms, then fitted to a one-phase exponential decay (Fig. 2d). Cas9 half-distances were 2–5 μm at 48 and 72 hours, which was less than the average cell radius (12 ± 3 μm), indicating no spreading. NANITE half-distances were 19 and 42 μm at 48 and 72 hours respectively, showing progressive radial spread of editing enzymes multiple cell lengths from initially transfected cells.

We next wondered whether NANITE worked in other cell types. We compared editing mediated by NANITE or Cas9 at the endogenous *B2M* locus in HepG2 liver cells, which are commonly used as an *in vitro* model for hepatocytes^19^. Edited cells in the NANITE condition averaged 238% of the transfected population compared to ~92% in the Cas9 condition (Supplementary Fig. 3a). Similar results were observed in C2C12 muscle cells (Supplementary Fig. 3b). These results show that NANITE can spread genome editing beyond transfected cells in multiple cell types.

### The specificity of NANITE can be tuned

**Figure 3.**
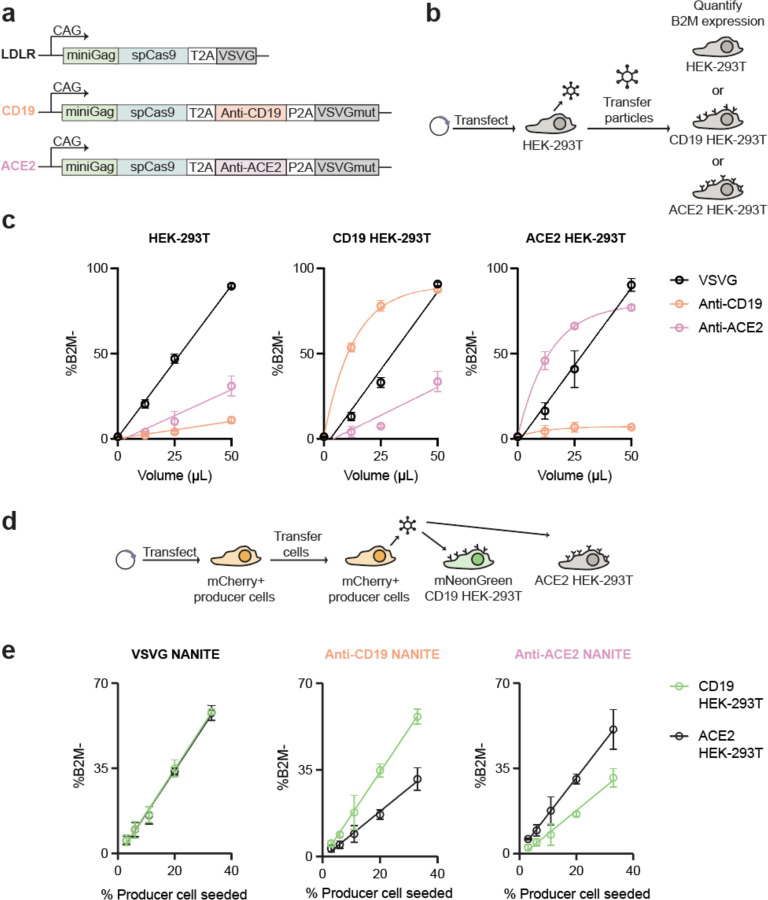
NANITE can be targeted. **(a)** Schematic of targeted NANITE designs, in which single-chain antibody fragments are included with a binding-deficient VSVG. **(b)** Experimental scheme for testing targeted NANITE designs. HEK-293T cells were transfected, vesicles were harvested, and then added to recipient cells. Schematics are not to scale. **(c)** Fraction of edited HEK-293T, CD19-expressing HEK-293T, or ACE2-expressing HEK-293T cells after incubation with VSVG (black), anti-CD19 (orange), or anti-ACE2 (pink) NANITE. VSVG-NANITE edited all cell types, whereas targeted NANITE was specific for cells expressing the specific receptor. Data are presented as mean ± SD of three replicates. **(d)** Experimental scheme for testing targeted NANITE designs *in situ*. Producer cells (mCherry^⁺^) were transfected with NANITE plasmids and co-cultured with 1:1 mixtures of CD19-expressing HEK-293T (mNeonGreen^⁺^) and ACE2-expressing HEK-293T cells. Editing at the *B2M* locus was quantified by flow cytometry 3 days after co-culture. **(e)** Fraction of edited CD19-expressing HEK-293T (green) or ACE2-expressing HEK-293T (black) cells after co-culture with producer cells expressing targeted NANITE constructs. VSVG-NANITE resulted in equivalent editing of both cell types, whereas targeted designs showed preferential editing of cells expressing their specific receptor. Data are presented as mean ± SD of three replicates.

Previous studies showed that EDV tropism can be tuned by incorporating single-chain antibody fragments (scFvs) with receptor-binding deficient fusogens^11,20,21^. We explored whether NANITE tropism could be similarly controlled by designing NANITE to display both scFvs and receptor-binding deficient VSVG (VSVGmut) simultaneously (Fig. 3a). CD19- and ACE2-binding scFvs were selected because they were validated targeting molecules whose receptors are not endogenously expressed in HEK-293T cells, enabling specificity testing without confounding background receptor expression^22,23^. We transfected HEK-293T cells with targeted NANITE plasmids and harvested supernatants for incubation with recipient cells either expressing or lacking the cognate receptor (Fig. 3b). Editing at the *B2M* locus was quantified by flow cytometry (Supplementary Fig. 4a, b). VSVG-NANITE edited all cell types equally (Fig. 3c, black), while Anti-CD19-NANITE and Anti-ACE2-NANITE showed up to 25-fold and 11-fold higher editing of cells expressing their matching receptors (Fig. 3c, orange and pink respectively), demonstrating that NANITE tropism can be engineered modularly.

We further wondered whether this strategy directed NANITE tropism *in situ.* We transfected mCherry-labelled producer cells with targeted NANITE plasmids and co-cultured them at different ratios (1:3 to 1:32) with a fifty-fifty mix of CD19-expressing HEK-293T (mNeonGreen+) and ACE2-expressing HEK-293T cells (Fig. 3d). Editing at the B2M locus in recipient cells was quantified by flow cytometry (gating shown in Supplementary Fig. 4c). We observed roughly 2-fold higher editing of cells when they expressed the matching receptor (Fig. 3e), while VSVG-NANITE edited both cell types equally. These results demonstrate that the *in situ* specificity of NANITE can be directed. Altogether our results show that EDVs with programmable tropism can be produced from a single plasmid.

Beyond controlling tropism using scFvs, we reasoned that because NANITE is ultimately a nucleic acid cargo, cell-type specificity could also be controlled at the transcriptional level using tissue-specific promoters. We replaced the CAG promoter with liver-specific TGB610, GFAP and LP1 promoters (Supplementary Fig. 5a) and transfected equimolar amounts into HEK-293T or HepG2 cells to assess NANITE activity as measured by BFP expression (Supplementary Fig. 5b)^24–26^. To compare the promoters across cell lines, NANITE activity was normalized to the CAG promoter within that cell type. The TGB610 promoter showed significantly higher activity in on-target HepG2 compared to off-target HEK293T cells, but with only 34% the activity of CAG (Supplementary Fig. 5c). Our results are concordant with previous studies showing a trade-off between promoter expression strength and specificity^27^. Nevertheless, these results show the potential to control NANITE expression through promoter engineering. Altogether, we were able to control the specificity of NANITE at multiple stages of deployment.

### NANITE amplifies editing efficiencies in vivo

Having shown that NANITE could amplify editing efficiencies *in vitro*, we next explored whether NANITE could amplify genome editing efficiencies *in vivo*. We chose to test NANITE for transthyretin (TTR) liver editing in mice, a well-characterized therapeutic target for transthyretin amyloidosis with validated sgRNAs and characterization assays^28,29^. We used the NANITE design with the CAG promoter and VSVG, as CAG showed the highest *in vitro* activity and VSVG naturally transduces hepatocytes via the low-density lipoprotein receptor (LDLR). We first tested using recently published lipid nanoparticles (LNPs) that contain a cGAS-STING inhibitor (composition in Supplementary Fig. 6a)^30^ to package and deliver plasmids *in vivo*. LNPs were ~109 nm in diameter with ~85% encapsulation efficiency (Supplementary Fig. 6b). NANITE-LNPs amplified editing *in vitro* (Supplementary Fig. 6c), but *in vivo* doses up to 25 µg (matching the highest dose previously reported^30^) produced no detectable transfection (Supplementary Fig. 6d, e). This prompted us to explore alternative *in vivo* plasmid delivery methods.

**Figure 4.**
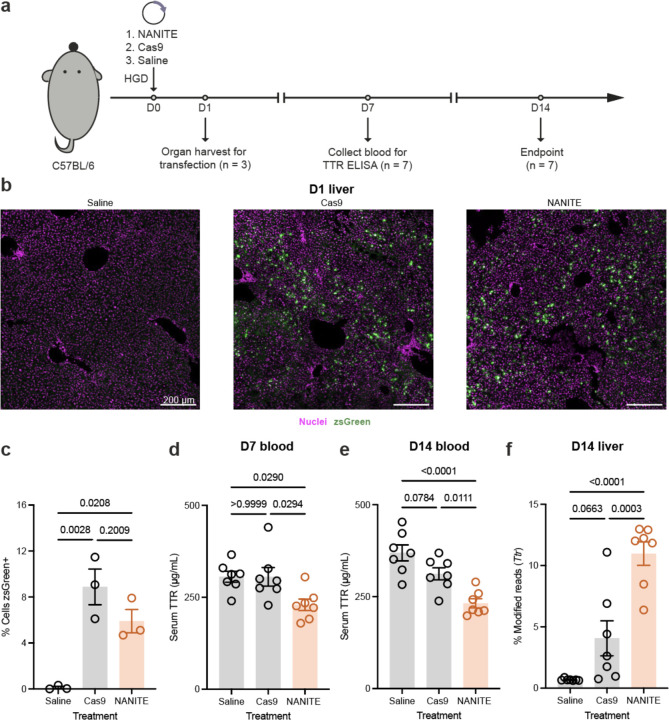
NANITE amplifies editing *in vivo.* **(a)** Experimental scheme for mouse experiments for editing the *Ttr* locus. Mice were hydrodynamically injected (HGD) with plasmids. Organs were collected 24 hours post-injection to quantify transfection efficiency (n = 3). Editing was monitored by TTR ELISA on blood collected on days 1, 7, and 14. Tissues were harvested at day 14 to assess editing and animal health (n = 7). **(b)** Representative immunofluorescence images of liver sections showing nuclei (magenta) and zsGreen (green). Scale bar as indicated. **(c)** Fraction of transfected cells in the liver. One field of view per mouse was quantified. No significant difference in the fraction of transfected cells was observed between Cas9 and NANITE conditions. **(d)** Serum TTR concentration 7 days post-administration. NANITE significantly reduced TTR levels. **(e)** Serum TTR concentration 14 days post-administration. NANITE significantly reduced TTR levels. **(f)** Next-generation sequencing of liver genomic DNA harvested 14 days post-injection. NANITE edited significantly more cells than saline or Cas9 groups. For (c–f), statistical significance was determined by one-way ANOVA with Tukey’s multiple comparison test. p-values are indicated. Data are presented as mean ± SEM (c: n = 3 mice; d–f: n = 7 mice).

We subsequently turned to hydrodynamic injection, a validated method for transiently delivering plasmids to liver cells^31,32^. Hydrodynamic injections have been explored in humans for liver gene therapy^32,33^. Our dose optimization showed that 20 pmol plasmid achieved maximum transfection (~5% of liver cells) (Supplementary Fig. 7a-c), consistent with published efficiencies for 10–15 kb plasmids^34^. To see if NANITE could amplify editing *in vivo,* we began our experiment by injecting either NANITE- or Cas9-expressing plasmids at this 20 pmol dose (Fig. 4a). We then quantified transfection efficiencies between groups to ensure they were comparable to allow comparison of editing efficiencies. Similar transfection efficiencies were observed between Cas9- and NANITE-treated mice at 24 hours, as assessed by zsGreen fluorescence imaging (Fig. 4b, c; 8 ± 3% and 6 ± 2%, respectively) and flow cytometry (Supplementary Fig. 8). We quantified transfection at 24 hours, because plasmid expression is known to be maximal at 24 hours in mice^35,36^. Western blot analyses of liver lysates confirmed NANITE protein production *in vivo*, albeit at lower levels than Cas9 likely due to its larger size (Supplementary Fig. 9). These results demonstrate that a similar fraction of cells were transfected with both plasmid types, but protein expression was higher in the Cas9-expressing group.

Since transthyretin is secreted into the bloodstream, we monitored the serum transthyretin concentrations using enzyme-linked immunosorbent assays (ELISAs). By day seven, NANITE-treated mice showed significantly reduced serum TTR levels (Fig. 4d), persisting to day 14 when NANITE mice had 60 ± 11 % the TTR concentration of saline controls (Fig. 4e). In contrast, Cas9-treated mice only showed numerically lower levels without statistical significance (85 ± 17%). Since serum TTR levels plateaued between days 7 and 14, we terminated the experiment at day 14. At the endpoint, next-generation sequencing revealed that NANITE treatment averaged 11 ± 3 % TTR editing. In contrast, Cas9-treated mice only averaged 4 ± 4 % TTR editing. (Fig. 4f). Notably, NANITE outperformed Cas9, despite Cas9 showing higher protein expression at comparable transfection rates. These results show that the elevated expression from Cas9 plasmids did not proportionally increase tissue-level editing, likely because excess editing enzymes were confined intracellularly. NANITE’s ability to spread editing enzymes between cells overcomes this limitation, enabling more efficient tissue-level editing. Immunohistochemistry and Western blotting detected no zsGreen or flag-tagged protein 14 days post-administration (Supplementary Fig. 10a-c), confirming transient expression. Altogether, these results demonstrate that transient expression of NANITE is sufficient to amplify editing efficiencies *in vivo*.

Lastly, we examined the safety of NANITE treatment. No significant off-target editing was detected at two predicted loci (Supplementary Fig. 11a-c). RT-qPCR did not detect elevated sgRNAs concentrations in serum from NANITE-treated mice compared to Cas9 controls (Supplementary Fig. 11d, e), confirming that NANITE activity was largely confined to the liver. Concomitantly, no editing occurred in ovaries (Supplementary Fig. 10f). All mice experienced an initial 5–10% weight loss post-injection, recovering by day 4, with no group differences (Supplementary Fig. 12a). Blinded histopathology scoring on the liver revealed no elevated steatosis, inflammation, fibrosis, mitosis, glycogen accumulation, necrosis, karyomegaly, bile duct inflammation, or mineralization in Cas9- or NANITE-treated mice (Supplementary Fig. 12b-m). To evaluate physiological function of the liver and other organs, we also performed blood biochemistry. Aspartate transaminase levels were elevated but remained within normal ranges in both Cas9- and NANITE-treated mice (Supplementary Fig. 13a). There were no significant differences in other markers (Supplementary Fig.13b-k). Taken together, these results show that NANITE amplified editing *in vivo* without detectable toxicity.

## Discussion

A major goal in genome editor delivery is ensuring that sufficient cells are transfected and edited to produce physiological effects. Realizing *in vivo* genome editing therapies has relied on engineering delivery vehicles to surpass these thresholds by reaching more cells. Here, we introduce a complementary paradigm to expand editing by enabling transient cell-to-cell spread of editing enzymes. Rather than directly administering purified EDVs or other VLPs^11–15^, we delivered their encoding plasmid. *In vivo* transfection of nucleic acids encoding EDVs roughly tripled genome editing compared with Cas9 RNP-only controls. NANITE reduced serum TTR levels *in vivo* by nearly 50%. Clinical studies have shown that a ~50% reduction in the disease-causing TTR could result in disease improvement and stabilization^37^. We were able to achieve clinically relevant levels of editing despite low initial transfection rates using NANITE. In contrast, Cas9 plasmids administered using the same method did not produce statistically significant reductions in serum TTR levels. Future work may further increase NANITE amplification by prolonging or increasing its expression *in vivo.*

A limitation of NANITE is that *in vivo* plasmid delivery is currently inefficient^30^. NANITE is currently encoded as a plasmid, because it requires a cassette expressing sgRNA to form SpCas9 RNPs. However, continued advances in DNA-delivery lipid nanoparticles^38^ and potential conversion to mRNA format could allow for compatibility with the plethora of lipid nanoparticles available for *in vivo* editing^39–42^. As NANITE is ultimately a nucleic acid cargo, we believe that it will be compatible with future nucleic acid delivery modalities (including LNPs, retroviral vectors and adenoviral vectors) and has the potential to multiply their effectiveness. We also believe that NANITE may be applicable to other therapeutic proteins.

More broadly, NANITE represents a conceptual shift in the delivery of therapeutic cargo. Rather than solely improving how cargo reaches cells, we can try to amplify therapeutic effects by spreading cargo beyond initially transfected cells. This amplification effectively lowers the fraction of cells that must be directly transfected to achieve a therapeutic effect. Critically, this could expand genome editing to previously intractable tissues, not by improving delivery vehicles themselves, but by making suboptimal delivery sufficient. Many tissues remain difficult to transfect at therapeutically relevant efficiencies with current technologies. NANITE could bridge this gap by amplifying modest transfection into meaningful therapeutic effects. In the future, we envision pairing tissue-tropic delivery vehicles with correspondingly targeted NANITE constructs to achieve tissue-specific editing amplification. By lowering effective dose requirements, NANITE could make genome editing more practical and accessible for treating human disease.

## Methods

### Cloning plasmids

Plasmids were cloned using HiFi assembly (New England Biolabs, #E2621X). DNA fragments for assembly were generated by PCR using Q5 PCR Master Mix (New England Biolabs, #M0492L) or ordered as gene blocks (Twist Bioscience or Integrated DNA Technologies). To create the NANITE plasmid, VSVG was amplified from pMD2.G (Addgene, #12259) using primers with appropriate overhangs (Integrated DNA Technologies). The VSVG and T2A fragments (Integrated DNA Technologies) were inserted downstream of Cas9 in pWN_U6-B2M-miniGag (Addgene, #228957). Other plasmids were cloned using a similar strategy. CRISPR-Cas9 spacer sequences are listed in Supplementary Table 1.

Plasmid and HiFi assemblies were transformed into Mach1 *E. coli* cells (Thermo Fisher Scientific, #C862003) rendered competent using the Mix & Go *E. coli* Transformation Kit (Zymo Research, #T3001). Mach1 were grown with appropriate antibiotic selection, then plasmids were extracted and purified using miniprep (Qiagen, #27104), midiprep (Zymo Research, #D4200), or maxiprep kits (Zymo Research, #D4202). For animal experiments, plasmids were extracted using the EndoFree Plasmid Maxi Kit (Qiagen, #12362) to minimize endotoxin contamination. Endotoxin levels were quantified using limulus amebocyte lysate assays (Thermo Fisher Scientific, #A39552S) prior to administration. All plasmids were verified by whole-plasmid sequencing (Plasmidsaurus).

### Cell culture

HEK-293T cells (gift from Dr. Melanie Ott, Gladstone Institutes) were seeded on 15-cm plates (Fisher Scientific, #FB012925) in DMEM (Corning, #10-013-CV) supplemented with 10 v/v% FBS (Avantor, #97068–085) and 1 v/v% penicillin/streptomycin (Thermo Fisher Scientific, #15140122). Cells were incubated at 37°C with 5% CO_₂_ and passaged as required. C2C12 cells (ATCC, #CRL-1772) were similarly cultured. HepG2 cells (ATCC, #HB-8065) were cultured in EMEM (ATCC, #30–2003) supplemented with 10 v/v% FBS and 1 v/v% penicillin /streptomycin. All cells were routinely tested for mycoplasma contamination (Stem Cell Core, Gladstone Institutes).

### Generating BFP-reporter cells

EF-1α promoter, blue fluorescent protein (BFP) reporter, and puromycin resistance fragments (Supplementary Fig. 1a) were cloned into a pLVX backbone using HiFi assembly to generate a lentiviral transfer plasmid. To produce lentiviral vectors, 4 million HEK-293T cells were seeded on 10-cm dishes (Corning, #35300). The following day, cells were transfected with 2.5 μg of transfer plasmid, 1 μg pMD2.G, and 10 μg psPAX2 using polyethylenimine (PEI; Polysciences, #23966) at a 3:1 ratio (PEI:DNA by mass). Excess plasmid was removed after 24 hours, and lentiviral vectors were harvested at 48 hours. HEK-293T cells (48,000 cells) were incubated with 0.5 mL lentiviral vectors and 8 μg/mL polybrene (MilliporeSigma, #H9268) for 48 hours, followed by selection in 2 μg/mL puromycin (InvivoGen, #ant-pr-1) for 48 hours.

To validate reporter cells, miniEDVs targeting either the BFP-reporter or TRAC locus were produced as previously published^13^, then 8–500 μL were incubated with 15,000 reporter cells in 24-well plates (Corning, #353047). Cells were trypsinized after 72 hours and stained with 1 μL Zombie NIR live-dead stain (BioLegend, #423106). BFP expression was quantified by flow cytometry on an Attune NxT Flow Cytometer (Thermo Fisher Scientific). Flow gating is shown in Supplementary Fig. 1d, e. Genomic DNA was harvested using QuickExtract reagent (Biosearch Technologies, #QE09050). The reporter locus was amplified by PCR (BFPreporter.rev and BFPreporter.for; sequences in Supplementary Table 2) and Sanger sequenced (Elim Biopharm). Insertion and deletion frequencies were calculated using the TIDE analysis webserver^43,44^. Cells were stored in liquid nitrogen for use in downstream experiments. Similar procedures were used to generate BFP-reporter C2C12 and HepG2 cells.

### Testing single plasmids encoding NANITE

To produce vesicles, 600,000 HEK-293T cells were seeded per well of a 6-well plate (Corning, #3516). The following day, cells were transfected with 2.2 μg NANITE plasmid or 2 μg pWN_U6-BFPreporter-miniGag and 0.2 μg pMD2.G as a positive control using PEI. Excess plasmid was removed after 24 hours. Vesicles were harvested 48 hours post-transfection as previously published^13^, and up to 50 μL was incubated with 7,500 BFP-reporter HEK-293T cells in 48-well plates (Corning, #3548). Cells were trypsinized after 72 hours and stained with Zombie NIR live-dead stain (BioLegend, #423106). BFP expression was quantified by flow cytometry on an Attune NxT Flow Cytometer. Flow gating is shown in Supplementary Fig. 1d, e.

For Western blotting of cell lysates, HEK-293T cells were harvested 48 hours post-transfection using RIPA lysis buffer (Thermo Fisher Scientific, #89900) with 1× protease inhibitors (Thermo Fisher Scientific, #78429). Lysates were centrifuged at >19,000 × g for 5 minutes to remove insoluble aggregates. Supernatants were stored at −80°C. Protein concentrations were determined using the BCA assay (Thermo Fisher Scientific, #23225). Samples normalized for protein concentration were mixed with 4× Laemmli sample buffer (Bio-Rad, #1610747) to a final 1× concentration with 1% (v/v) 2-mercaptoethanol (MilliporeSigma, #M3148), then denatured at 90°C for 5 minutes. Samples were run on SDS-PAGE gels (Bio-Rad, #5678094) for 2 hours at 120 V at 4°C. Proteins were transferred to 0.2 μm nitrocellulose membranes using a Trans-Blot Turbo Transfer Pack (Bio-Rad, #1704159) at 2.5 A and 25 V for 7 minutes using a Trans-Blot Turbo Transfer System (Bio-Rad). Membranes were blocked with 5 w/v% non-fat dry milk (Lab Scientific bioKEMIX, #M0841) in 1× TBS-T for at least 2 hours at room temperature. 1×TBS-T consists of 10 mM Tris (Corning, #46-030-CM), 150 mM NaCl (Thermo Scientific, #J21618-A9), and 0.05 v/v% Tween-20 (Fisher, #BP337). Membranes were incubated overnight at 4°C with anti-FLAG antibody (MilliporeSigma, #F1804, 1:1,000), anti-VSVG antibody (Kerafast, #Kf-Ab01401–23.0, 1:1,000), or anti-GAPDH antibody (Santa Cruz Biotechnology, #sc-365062, 1:1,000) in blocking solution as appropriate. Membranes were washed 3–5 times for 5 minutes each with 1× TBS-T, then incubated with the appropriate secondary antibody (goat anti-mouse, Invitrogen, #62–6520, 1:1,000; or goat anti-rabbit, Invitrogen, #656120, 1:1,000) in blocking solution for 1 hour at room temperature. Membranes were washed 3–5 times and developed using chemiluminescent substrate (Thermo Fisher Scientific, #34580). Images were acquired using a ChemiDoc MP imaging system (Bio-Rad). The unprocessed blot is shown in Supplementary Fig. 14.

### Protease protection assay

Protease protection assays were performed as published^6^. Vesicles were produced in HEK-293T cells and harvested after ~72 hours as described above. Samples were incubated on ice for 30 minutes alone or with 1 v/v% Triton X-100 (Sigma-Aldrich, #X100), 10 μg/mL proteinase K (New England Biolabs, #P8107S), or both. PMSF (Sigma-Aldrich, #P7626) was added to a final concentration of 5 mM to inhibit the proteinase K and incubated for 5 minutes on ice. Laemmli buffer was added to a final concentration of 20 v/v% and samples were heated at 98°C for 5 minutes. Samples were run on SDS-PAGE gels (Bio-Rad, #4561095) and transferred to PVDF membranes (Bio-Rad, #1620177) at 90 V and 4°C for 1 hour in transfer buffer. The transfer buffer consists of 25 mM Tris (Avantor, #4099–06), 192 mM glycine (Bio-Rad, #1610724), and 20 v/v% methanol (VWR, #BDH1135). Membranes were blocked for 1 hour at room temperature on an orbital shaker in blocking buffer consisting of 1× PBS (Gibco, #14190) with 0.1% Tween-20 (Sigma-Aldrich, #P7949) saturated with non-fat dry milk (Bio-Rad, #1706404XTU). Membranes were washed four times with 1྾PBS containing 0.1% Tween-20 (PBS-T), then incubated overnight at 4°C in blocking buffer with anti-FLAG antibody (Sigma-Aldrich, #F1804, 1:2,000). Membranes were washed four times with PBS-T, then incubated with secondary antibody (LI-COR, #926–68070, 1:20,000) for 1 hour at room temperature. The membranes were then washed four times with PBS-T, and imaged on a LICOR imager (LICORBio). The unprocessed blot is shown in Supplementary Fig. 14.

### In situ editing with NANITE

BFP-reporter HEK-293T cells (10,000 cells/well) were seeded in 96-well plates (Corning, #3596). The following day, cells were transfected with 15–100 ng NANITE or Cas9 control plasmids using PEI at a 3:1 ratio (PEI:DNA by mass). The media were replaced after 24 hours to remove excess plasmid. Flow cytometry was performed as described above, with example gating shown in Supplementary Fig. 1d and Fig. 2B. Experiments in HepG2 and C2C12 cells were performed similarly using jetPRIME (Sartorius, #101000015) and Cell Avalanche (EZ Biosystems, #EZT-C2C1–1) transfection reagents, respectively. To quantify B2M expression in HepG2 cells, cells were stained with 5 μL APC anti-human β2-microglobulin (BioLegend, #316312) and 1 μL Zombie UV live-dead stain (BioLegend, #423108). B2M expression was quantified on an Attune NxT Flow Cytometer.

To visualize NANITE spread, 20,000 BFP-reporter cells per well were seeded into polylysine-coated (Thermo Fisher Scientific, #A3890401) chamber slides (Thermo Fisher Scientific, #154526). Cells were transfected with NANITE or Cas9 plasmids. Media were replaced after 24 hours to remove excess plasmid. At each timepoint, cells were fixed with 4% buffered formaldehyde (MilliporeSigma, #1.00496), then permeabilized with 0.1 v/v% Triton X-100 (Fisher Scientific, #BP151–100) in 1× PBS. Fixed permeabilized cells were washed with 5 w/v% bovine serum albumin (MilliporeSigma, #A9647) in 1× PBS. Nuclei were stained with RedDot 2 far-red nuclear stain (Biotium, #40061) for 10 minutes at room temperature. Stained cells were washed three times with 5 w/v% bovine serum albumin in 1× PBS before mounting with antifade mounting medium (Vector Laboratories, #VECTH170010). Cells were imaged with identical settings using a Zeiss LSM880 confocal microscope (Gladstone Histology and Light Microscopy Core). Two biological replicates per condition were imaged, with at least three fields of view each. Nuclei were segmented and classified as zsGreen- or BFP-positive using CellProfiler 4.2.8^18^. The distance from each BFP nucleus to the nearest zsGreen nucleus was measured and binned into histograms. Frequency distributions were fitted to a one-phase exponential decay in GraphPad Prism v.10.6.1 to determine half-distances.

### Targeting NANITE

Anti-CD19 and anti-ACE2 single-chain variable fragments (scFvs) were designed as previously described^11,13,23^ and inserted upstream of VSVG in the NANITE plasmid with a P2A linker. Mutations in the VSVG low-density lipoprotein receptor-binding region (K47Q, R354A) were introduced using appropriate primers and HiFi assembly. Plasmid sequences were verified and vesicles were produced as described above. Vesicles (12.5–50 μL) were incubated with 10,000 wild-type, CD19-expressing, or ACE2-expressing HEK-293T cells. Editing at the *B2M* locus was quantified 96 hours post-incubation by staining cells with 5 μL APC anti-human β2-microglobulin and 1 μL Zombie UV live-dead stain. B2M expression was quantified on an Attune NxT Flow Cytometer. The example flow gating strategy is shown in Supplementary Fig. 4a, b.

For *in situ* experiments, NANITE plasmids were transfected into mCherry-expressing HEK-293T cells. The following day, transfected cells (313–5,000 cells) were mixed with 5,000 CD19-expressing HEK-293T cells (mNeonGreen^⁺^) and 5,000 ACE2-expressing HEK-293T cells in a 96-well plate. After 72 hours of co-culture, B2M expression was quantified by flow cytometry as described above. The gating strategy is shown in Supplementary Fig. 4c.

TGB610^24^, GFAP^25,45^, and LP1^26^ promoter sequences were purchased as gene fragments from Twist Bioscience. The CAG promoter in the NANITE plasmid was replaced with each of these promoters using HiFi assembly. Sequence-verified plasmids were transfected into BFP-reporter HEK-293T or HepG2 cells at 5 fmol and 24 fmol using PEI and jetPRIME reagents, respectively. An equimolar amount of plasmid was used to ensure cells received the same number of plasmids independent of the plasmid size. The following day, media were replaced to remove excess plasmid. BFP expression was quantified by flow cytometry 72 hours post-transfection as described above. To compare NANITE activity between cell lines, BFP expression was normalized to the CAG promoter condition.

### Administering NANITE in vivo

All mouse protocols were approved by the Institutional Animal Care and Use Committee (IACUC) at UCSF. Mice were housed in a barrier animal facility with standard 12-hour light/dark cycles at the Gladstone Institutes. All experiments were performed with 6-12-week-old female C57BL/6 mice (The Jackson Laboratory, #000664). Female mice were used to enable assessment of potential germline editing in ovaries, a key safety consideration for genome editing therapies with systemic exposure.

We initially packaged NANITE plasmids into lipid nanoparticles (LNPs) following previously published protocols^30^. Briefly, 50 mol% SM-102 (Cayman Chemical, #33474), 38.5 mol% cholesterol (MilliporeSigma, #C8667), 10 mol% DSPC (Avanti Research, #850365), and 1.5 mol% DMG-PEG2000 (Avanti Research, #880151) were mixed in anhydrous ethanol (Decon Laboratories, #V1016). The final lipid concentration of 12.5 mM. 9(10)-Nitrooleic acid (Cayman Chemical, #33896) was then added at 20 mol% of total lipid. Plasmids were diluted in 50 mM citrate buffer (pH 4) to a final concentration of 0.065 mg/mL. LNPs were formulated using the NanoAssemblr Ignite (Cytiva) with the following parameters: flow rate ratio of 1:3 (ethanol:aqueous), flow rate of 6 mL/min, and waste volumes of 0.15 mL and 0.05 mL at the beginning and end, respectively. LNPs were washed four times using 10 kDa Amicon Ultra centrifugal filters (MilliporeSigma, #UFC8010) using 1×PBS. Hydrodynamic diameters and polydispersity indices were measured using a BeNano 180 Zeta Max (Bettersize). Encapsulation efficiency was determined using Quant-iT PicoGreen assays (Thermo Fisher Scientific, #P7589) as previously described ^30^. For functional testing, LNPs (6–100 ng plasmid DNA) were incubated with BFP-reporter HEK-293T cells. Media were replaced the following day to remove excess LNPs. BFP and zsGreen expressions were quantified by flow cytometry as above. Following characterization, 0, 12.5, or 25 μg LNP-encapsulated plasmid was injected into C57BL/6 mice, matching the highest doses safely tested previously^30^. Transfection efficiency was assessed the following day, as plasmid expression peaks at approximately 24 hours post-transfection (33, 34). Livers (either the left or right lateral lobe) were harvested and fixed for 24 hours in 4% buffered formaldehyde at 4°C, then transferred to 20 w/v% sucrose (Fisher Scientific, #BP220) in 1×PBS for 3 days. Livers were mounted in cryoblocks using Tissue-Plus O.C.T. Compound (Fisher Scientific, #23-730-571). Sections (20 μm) were cut using a Leica CM 3050 S cryostat, placed on microscope slides (Fisher Scientific, #22-037-246), and stored at −80°C. Sections were blocked with 1× Animal-Free Blocker (Vector Laboratories, #SP-5030) containing 0.03 v/v% Triton X-100 and 0.05 w/v% sodium azide (MilliporeSigma, #71289) for 1 hour at room temperature, then stained with DAPI (MilliporeSigma, #10236276001) for 30 minutes. Sections were coverslipped (Avantor, #48393–060) with Fluoromount-G (SouthernBiotech, #0100–01) and imaged using a Stellaris 5 confocal microscope (Leica) with identical settings across samples. No zsGreen fluorescence was detected, indicating no observable transfection.

We subsequently administered plasmids via hydrodynamic injection following UCSF standard protocols. For dose optimization studies, 15–34 pmol NANITE plasmid in saline (Teknova, #S5825) was injected at volumes equivalent to 10% of mouse body weight^31,32,34,46,47^. Transfection efficiency was assessed as above. The fraction of zsGreen-positive cells was quantified using CellProfiler by segmenting nuclei and zsGreen regions. Nuclei were masked with zsGreen regions to identify positive cells. The percentage of zsGreen-positive cells was calculated by dividing zsGreen-positive nuclei by total nuclei per field of view. For visualization, maximum and average intensity projections were applied to the DAPI and zsGreen channels, respectively, with identical thresholds across all images within an experiment.

To compare Cas9 and NANITE plasmids *in vivo* for *Ttr* editing, both were administered at 20 pmol. Equimolar doses were used to ensure animals received equivalent plasmid copy numbers regardless of plasmid size. Body weights were recorded daily. Blood was collected from the submandibular vein at 1, 7, and 14 days post-injection using sterile lancets (Braintree Scientific, #GR 5MM) into capillary blood collection tubes (Sarstedt, #20.1280.100). Blood was kept on ice. To obtain serum, blood was centrifuged at 1,600 × g for 10 minutes at 4°C. Serum was collected and stored at −80°C until analysis. A sub-cohort of mice was euthanized 24 hours post-injection to quantify transfection, and remaining mice were euthanized at day 14. Tissues were harvested at each endpoint. All procedures were performed according to our IACUC-approved protocol.

### Quantifying NANITE, Cas9 and zsGreen expression in vivo

Transfection efficiency was quantified 24 hours post-injection by imaging, flow cytometry, and Western blotting of liver lysates. ZsGreen expression was quantified by imaging as outlined above. One liver section per mouse was analyzed. Flow cytometry on liver cells was performed following previously published protocols^48,49^. Fresh liver tissue (~0.35 g) was minced using a sterile blade and digested with 50 U collagenase type I (Thermo Fisher Scientific, #17018029) in 1 mL Hanks’ balanced salt solution (HBSS; Thermo Fisher Scientific, #14175145) supplemented with 5 mM CaCl_₂_ (MilliporeSigma, #442909) at 37°C for 45 minutes with gentle shaking (300 rpm). The digested solution was filtered through a 70-μm cell strainer (Fisher Scientific, #22-363-548) and quenched with 2 mL HBSS supplemented with 2 v/v% FBS. Cell suspension (1 mL) was centrifuged at 500 × g for 5 minutes at 4°C to obtain a cell pellet. Hepatocytes were stained with 5 μL anti-CD95 antibody (BioLegend, #152620) and 1 μL Zombie NIR live-dead stain in HBSS for 30 minutes at 4°C. Cells were pelleted by centrifugation and washed with HBSS supplemented with 2 v/v% FBS. ZsGreen expression was quantified by flow cytometry on an Attune NxT Flow Cytometer. Gating is shown in Supplementary Fig. 8a, b.

For Western blot analysis of Cas9 and NANITE expression in liver lysates, ~20 mg liver tissue (from the left lateral, right lateral, and caudate lobes) was placed into 2 mL tubes prefilled with 1.4 mm ceramic beads (Omni International, #19–627) and 500 μL of RIPA lysis buffer with 1× protease inhibitors. Samples were kept on ice throughout processing. Tissue was homogenized using a BeadBug 6 (Benchmark Scientific) at 4,060 rpm for 9 seconds followed by 30 seconds of rest, repeated for 5 cycles. Lysates were centrifuged at >21,000 × g for 10 minutes at 4°C to remove debris. Supernatants were stored at −80°C until further processing. Samples were run on SDS-PAGE gels and transferred to 0.2 μm nitrocellulose membranes as described above. Membranes were blocked overnight at 4°C with 5 w/v% non-fat dry milk and 0.02 w/v% sodium azide (MilliporeSigma, #S2002–100G) in 1× TBS-T. Anti-FLAG antibody (MilliporeSigma, #F1804, 1:1,000) and anti-β-actin antibody (Cell Signaling, #4967, 1:1,000) in blocking solution were incubated with membranes overnight at 4°C. The anti-β-actin antibody was centrifuged at >19,000 × g for 10 minutes at 4°C to pellet protein aggregates prior to use. Membranes were washed 3–5 times for 5 minutes each with 1× TBS-T, then incubated with the appropriate secondary antibody (goat anti-mouse, Invitrogen, #62–6520, 1:1,000; or goat anti-rabbit, Invitrogen, #656120, 1:1,000) in blocking solution for 1 hour at room temperature. Membranes were washed 3–5 times and developed using chemiluminescent substrate (Thermo Fisher Scientific, #34580 or #34096). Images were acquired using a ChemiDoc MP imaging system. Raw Western blot images are shown in Supplementary Fig. 14. Imaging and Western blot analyses were repeated identically for liver samples harvested at day 14 to assess persistence of expression. Raw Western blot images are shown in Supplementary Fig. 14.

### Serum analyses

Circulating prealbumin levels were quantified using the Mouse Prealbumin SimpleStep ELISA Kit (Abcam, #ab282297) according to the manufacturer’s instructions. Briefly, serum was diluted 1:100,000 in the kit dilution buffer. Samples or standards (50 μL) and 50 μL of antibody mix were added to assay plates and incubated for 1 hour at room temperature on an orbital shaker. Wells were washed three times with 300 μL 1× wash buffer, then given a final wash with 150 μL 1× wash buffer. TMB developing solution (100 μL) was added to each well, and plates were incubated for >15 minutes at room temperature to allow color development. The reaction was stopped by adding 100 μL of stop solution. Absorbance (λ = 450 nm) was measured immediately using a Spark 20M plate reader (Tecan).

We also quantified the serum sgRNA concentrations at 1 and 14 days post-injection. Serum samples were diluted 1:100 in 1× DNA/RNA Shield DirectDetect reagent (Zymo Research, #R1400). Standard curves were generated using unmodified *Ttr* sgRNA (Integrated DNA Technologies) in untreated mouse serum diluted 1:100 in 1× DNA/RNA Shield DirectDetect. Samples or standards (4 μL) were combined with 2.5 μL of Luna Universal qPCR Master Mix (New England Biolabs, #M3003), 2 μL of reverse-transcription primer, 0.5 μL of probes (Custom TaqMan Small RNA Assay CTAACJP, Thermo Fisher Scientific), and 1 μL of nuclease-free water (Thermo Fisher Scientific, #AM9937). Reactions were assembled in 384-well plates (Applied Biosystems, #A36931) and sealed with optical adhesive film (Applied Biosystems, #4311971). RT-qPRC was run on a QuantStudio 5 Real-Time PCR System (Applied Biosystems) with the following settings: carryover prevention (25°C, 30 seconds), reverse transcription (55°C, 15 minutes), initial denaturation (95°C, 1 minute), and 45 cycles of denaturation (95°C, 10 seconds) and extension (60°C, 60 seconds) with a plate read.

To assess potential toxicity, blood chemistry analysis was performed on day 14 serum samples. This timepoint was selected to capture cumulative effects. Serum was submitted to the UC Davis Comparative Pathology Laboratory for analysis using the Chem-11 panel, measuring alanine aminotransferase, aspartate aminotransferase, alkaline phosphatase, total bilirubin, calcium, phosphorus, blood urea nitrogen, creatinine, glucose, total protein, and albumin.

### Next generation sequencing

Genomic DNA was extracted from fresh tissue using the Monarch Spin gDNA Extraction Kit (New England Biolabs, #T3010). Fresh liver tissue (~20 mg from the left lateral, right lateral, and caudate lobes) or ovaries were placed into 200 μL tissue lysis buffer with 3 μL proteinase K. Tissues were minced in lysis buffer using sterile scissors, then incubated at 56°C with agitation (1,400 rpm) for 45 minutes. Tissue debris was removed by centrifugation at >12,000 × g for 3 minutes, and 3 μL RNase A was added to the supernatant. Samples were incubated for 5 minutes at 56°C with agitation (1,400 rpm). Genomic DNA binding and column purification were performed according to the manufacturer’s protocol.

Target regions were amplified by PCR using Q5 High-Fidelity DNA Polymerase (New England Biolabs, #M0492) and primers listed in Supplementary Table 2. Two potential off-target sites were predicted using Cas-OFFinder 2.4.1, prioritizing sites with NGG PAMs allowing up to three mismatches with one DNA or RNA bulge^50^. Amplicons were column-purified using the QIAquick PCR Purification Kit (Qiagen, #28104) following the manufacturer’s protocol. Amplicon size and purity were verified by agarose gel electrophoresis, and concentration was determined using the Qubit dsDNA Quantification Assay (Thermo Fisher Scientific, #Q32854). Amplicons were submitted for Amplicon-EZ sequencing (~50,000 reads per sample; Genewiz). Indel frequencies were quantified using CRISPResso2 with default parameters for an NHEJ experiment as previously published^51^.

### Liver histopathology

Median or lateral lobes were harvested and fixed in 5 mL 4% buffered formaldehyde at 4°C for 24 hours. Following fixation, tissues were transferred to 70 v/v% ethanol and stored at 4°C until submission to the Gladstone Histology and Light Microscopy Core for processing. Liver lobes were placed in cassettes, processed, and embedded in paraffin. Sections were mounted onto glass slides and stained with hematoxylin and eosin (H&E). Slides were imaged using an Axioscan 7 slide scanner (Zeiss) at 20× magnification. Whole liver sections were scored for signs of hepatotoxicity by a licensed pathologist in a blinded manner (Histowiz). One section was scored per mouse.

### Software and Statistics

Flow cytometry data were analyzed using FlowJo 10.10.0 as indicated. Images were processed for visualization in (Fiji Is Just) ImageJ 2.16.0/1.54p. Image analyses were performed in CellProfiler 4.2.8 or (Fiji Is Just) ImageJ 2.16.0/1.54p as indicated. Cas-OFFinder 2.4.1 was used to predict off-target sites for genome editing. CRISPResso2 was used to analyze sequencing data and determine editing efficiencies. Data were plotted in GraphPad Prism v.10.6.1 or Microsoft Excel v.16.104. Statistical significance was determined using GraphPad Prism v.10.6.1 as indicated. Figures were created using Adobe Illustrator 28.4.1.

## Supplementary Material

Supplementary Figures 1–14 and Supplementary Table 1 – 2.

## Data Availability

Plasmids are available from Addgene. All other data and materials are available from the authors upon request.

## Code availability

Bespoke code was not used in this work.
